# Clinical, Social, and Healthcare Factors Associated with Nutritional Status in Amyotrophic Lateral Sclerosis: A Multidimensional Approach

**DOI:** 10.3390/biomedicines14081838

**Published:** 2026-08-15

**Authors:** Diogo Sousa-Catita, Paulo Mascarenhas, Cátia Oliveira, Miguel Grunho, Filipe Gonçalves, Jorge Fonseca

**Affiliations:** 1 Aging Lab, Egas Moniz Center for Interdisciplinary Research (CiiEM), Egas Moniz School of Health & Science, 2829-511 Almada, Portugal; pmascarenhas@egasmoniz.edu.pt (P.M.); miguelgrunho@gmail.com (M.G.); jorgedafonseca@gmail.com (J.F.); 2 Residências Montepio, Serviços de Saúde, S.A., Rua Julieta Ferrão No. 10, 5th Floor, 1600-131 Lisbon, Portugal; 3 APELA—Portuguese Association of Amyotrophic Lateral Sclerosis, 4200-227 Porto, Portugal; filipe.goncalves@apela.pt; 4 Egas Moniz Center for Interdisciplinary Research (CiiEM), Egas Moniz School of Health & Science, 2829-511 Almada, Portugal; 5 GENE—Artificial Nutrition Team, Department of Gastroenterology, Hospital Garcia de Orta, 2805-267 Almada, Portugal; sofi.doliveira@gmail.com; 6 Department of Neurology, Hospital Garcia de Orta, 2805-267 Almada, Portugal

**Keywords:** amyotrophic lateral sclerosis, nutritional status, GLIM criteria, MNA, multidimensional vulnerability, multidisciplinary care

## Abstract

**Background:** Amyotrophic lateral sclerosis (ALS) is a neurodegenerative disease where malnutrition significantly worsens clinical outcomes. We performed a multidimensional analysis to identify clinical, social, and healthcare factors associated with nutritional vulnerability in a real-world setting. **Methods:** This cross-sectional study included 97 adults with ALS recruited through a national association (APELA) and a hospital-based outpatient consultation (ANOC). Nutritional status was assessed using the Mini Nutritional Assessment (MNA^®^), Body Mass Index (BMI), and Global Leadership Initiative on Malnutrition (GLIM) criteria. Associations with total MNA^®^ scores were evaluated using a prespecified multivariable linear model with HC3 robust standard errors and bootstrap validation to assess inferential stability. **Results:** Malnutrition prevalence was high, with 42.3% of participants classified as moderately malnourished and 43.3% as severely malnourished under GLIM criteria. Mean MNA^®^ score was 19.69 (SD 4.53). In the adjusted model, ANOC recruitment was associated, after adjustment, with a 4.477-point lower MNA^®^ score compared with APELA (HC3 95% CI −7.071 to −1.884; p<0.001). Other sociodemographic and clinical factors yielded imprecise estimates. Although explanatory power was modest (Adjusted $R^{2}=0.067$; optimism-corrected $R^{2}=-0.005$), the recruitment setting remained a robust correlate across sensitivity analyses. **Conclusions:** Recruitment setting emerged as the most consistent adjusted correlate of MNA^®^ scores, likely reflecting differences in clinical case mix and referral pathways in the Portuguese healthcare context. These findings highlight nutritional vulnerability in ALS as a multifaceted construct that should be interpreted in relation to both clinical and healthcare-contextual factors. This study supports considering systemic factors and the need for longitudinal research that incorporates comprehensive functional and social metrics to optimize multidisciplinary nutritional interventions.

## 1. Introduction

Amyotrophic lateral sclerosis (ALS), also known as Lou Gehrig’s disease, is a rapidly progressive and fatal neurodegenerative disorder characterized by degeneration of upper and lower motor neurons in the brain and spinal cord. Clinically, it presents with progressive muscle weakness, spasticity, and eventual paralysis, with respiratory failure typically leading to death within 3 to 5 years of symptom onset [1]. Despite growing scientific efforts, the etiology of ALS remains largely unknown, and disease-modifying treatments are limited [2]. Nutritional status has long been recognized as a prognostic factor for survival in ALS [3].

Although ALS is relatively rare, affecting an estimated 2 to 5 per 100,000 people annually and resulting in approximately 140,000 new cases worldwide each year, its rapid progression imposes a significant physical, emotional, and economic burden on patients and caregivers [4]. Dysphagia and hypermetabolism accelerate weight loss, while the need for ventilatory support, gastrostomy, and home adaptations introduces substantial healthcare costs [5,6]. Caregiver burden, reduced work capacity, and psychological distress further contribute to the overall disease burden.

Nutrition is central to ALS management. The dual burden of reduced intake and increased energy expenditure places patients at high risk of malnutrition, which has been independently associated with faster functional decline and reduced survival [3]. Early, individualized nutritional assessment, tailored protein-energy feeding, and timely initiation of enteral feeding are essential components of care. A substantial proportion of patients require enteral feeding during advanced stages of the disease. Unfortunately, PEG feeding is often initiated late, after significant nutritional decline, due to a lack of timely nutritional planning [7]. Recent guidelines from the European Society for Clinical Nutrition and Metabolism (ESPEN) provide evidence-based recommendations for nutritional support in neurodegenerative diseases, including ALS [8].

Nutritional management is a recognized pillar of multidisciplinary ALS care and has been linked to improved quality of life in observational studies [9]. Clinical and nutritional vulnerability may also vary with broader contextual factors, including socioeconomic conditions, housing adequacy, caregiver availability, communication capacity, referral pathways, and access to specialized services. The independent associations of these factors require evaluation in appropriately specified models.

Recent literature supports multidisciplinary, person-centered models that integrate medical, nutritional, functional, and social domains in ALS care [10]. However, a gap remains in the literature on how these domains interact in real-world contexts, particularly outside controlled clinical settings. Most studies emphasize clinical progression and metabolic markers, with limited evaluation of the relationship between nutritional vulnerability and the broader clinical and healthcare context.

ALS care extends beyond clinical nutrition. Patients may face social and systemic barriers that affect their ability to maintain nutritional health and access care, including constraints related to socioeconomic circumstances, healthcare coverage, the home environment, caregiver availability, and geographic proximity to multidisciplinary services. These factors were therefore characterized in the present study, although the cross-sectional design cannot establish their causal effects.

Moreover, understanding nutritional risk in ALS should not be limited to identifying underweight status or reduced intake. Nutritional vulnerability may reflect functional dependence, communication impairment, psychosocial stress, and structural barriers to care. Rather than a purely biological consequence of disease progression, nutritional status in ALS may be conceptualized as a multidimensional construct reflecting interactions among clinical, functional, psychosocial, and healthcare-related factors. Instruments that capture this complexity are needed to guide both individualized care and health policy planning. Comprehensive assessments that extend beyond clinical data to include social and environmental determinants can offer deeper insight into how malnutrition develops and persists in ALS and how it can be prevented. In this context, the Mini Nutritional Assessment (MNA^®^) may be particularly useful because its total score incorporates nutritional, anthropometric, functional, psychological, clinical, and self-perceived health domains. Therefore, in the present study, the MNA^®^ was interpreted as an integrative measure of broad nutritional and functional vulnerability rather than as a purely nutritional or metabolic outcome.

This study aimed to characterize the nutritional status of individuals living with amyotrophic lateral sclerosis (ALS) and to explore clinical, social, and healthcare-related factors associated with broad nutritional vulnerability. Specifically, we sought to:

1. Assess nutritional status and broad nutritional vulnerability using complementary measures, including the total MNA^®^ score, BMI, and a study-specific operationalization of the GLIM criteria.
2. Describe clinical, functional, social, environmental, and healthcare-related characteristics in the overall cohort and selected clinical and nutritional characteristics by recruitment setting.
3. Examine the adjusted associations between the total MNA^®^ score and a clinically defined set of variables, including recruitment source, age, sex, ALS phenotype, time since diagnosis, comorbidity burden, education, and respiratory support.

By examining nutritional status alongside selected clinical and healthcare-related characteristics, this study aimed to advance a more precise understanding of nutritional and functional vulnerability in ALS. Given the observational and cross-sectional design, the analyses were intended to identify adjusted associations rather than causal effects and were not intended to develop an individual-level prediction tool.

## 2. Materials and Methods

### 2.1. Patients

The study included consecutive adults with amyotrophic lateral sclerosis (ALS) recruited between January 2024 and January 2025 through the Portuguese ALS Association (APELA) centres in Lisbon and Porto and the hospital-based Artificial Nutrition Outpatient Consultation (ANOC). ALS diagnosis was confirmed by a neurologist according to the revised El Escorial [11] or Awaji criteria [12], supported by the clinically indicated diagnostic work-up to exclude alternative diagnoses.

Participants were eligible if they were aged ≥ 18 years, had a confirmed diagnosis of ALS, and provided written informed consent. When necessary, informal caregivers assisted with data collection.

### 2.2. Questionnaire

Sociodemographic, clinical, and nutritional information was collected using a purpose-built structured questionnaire comprising more than 50 items, integrating validated instruments with study-specific questions tailored to ALS. The Portuguese version is provided in Appendix A. Data were collected at the Lisbon and Porto APELA centres and at the hospital-based Artificial Nutrition Outpatient Consultation (ANOC); the two APELA centres were combined as a single recruitment setting for analysis.

All questionnaires were administered during routine outpatient consultations by trained healthcare professionals using an interview format, with caregiver support as needed. To ensure data accuracy, responses were cross-checked against clinical records where available.

#### 2.2.1. Sociodemographic and Clinical Features

Sociodemographic characteristics included age (centered at 65 years), sex, and education level. Education was consolidated into ≤9 years versus ≥12 years for modeling to address sparse distribution at higher education levels. Employment status and region of residence were also recorded for descriptive purposes.

Clinical features included ALS onset phenotype (bulbar vs. non-bulbar, with spinal and other non-bulbar presentations combined) and comorbidity count. Respiratory support (yes/no) was recorded as an indicator of disease severity. Time since ALS diagnosis was calculated in days from the month and year of diagnosis, using the first day of the month as a convention, to the common cut-off date of 30 December 2024. For the primary adjusted model, this interval was log_2_-transformed to address right skew; the coefficient represents the change in total MNA^®^ score per doubling of the diagnosis-to-cut-off interval.

#### 2.2.2. Nutritional Assessment and Vulnerability

Nutritional status was assessed using the total Mini Nutritional Assessment (MNA^®^) score [13], which integrates screening and full-assessment components. In this study, 33 participants were younger than 65 years, and 64 were aged 65 years or older. Although the MNA^®^ was originally developed for older adults, its application to characterize nutritional risk in mixed-age adult cohorts with neurodegenerative diseases, including ALS, has been documented [14]. The total MNA^®^ score was utilized as a composite measure of broad nutritional vulnerability, reflecting the instrument’s integration of physical, functional, and self-perceived health domains. To evaluate the robustness of the primary findings, a prespecified sensitivity analysis was conducted by repeating the multivariable model exclusively among participants aged ≥65 years (*n* = 64).

#### 2.2.3. GLIM Criteria for Malnutrition Diagnosis and Staging

The Global Leadership Initiative on Malnutrition (GLIM) criteria [15] were operationalized to classify and stage malnutrition. A GLIM diagnosis required the presence of at least one phenotypic criterion and one etiologic criterion.

Phenotypic criteria included nonvolitional weight loss, low body mass index (BMI), and reduced muscle mass. Weight loss was calculated as the difference between current body weight and self-reported body weight from six months prior to assessment. Moderate weight loss was defined as a 5–10% reduction over the preceding six months, whereas severe weight loss was defined as a reduction exceeding 10% over the same period. Low BMI was defined as <20 kg/m^2^ for participants aged <70 years and <22 kg/m^2^ for those aged ≥70 years. Severe BMI thresholds were defined as <18.5 kg/m^2^ and <20 kg/m^2^, respectively, in accordance with GLIM guidance [15].

As instrumental body-composition measurements, including bioelectrical impedance analysis and dual-energy X-ray absorptiometry, were unavailable, reduced muscle mass was operationalized using calf circumference (CC) as a pragmatic anthropometric proxy, consistent with GLIM guidance for settings in which technical measurements are not feasible [16]. A CC < 31 cm was used to define reduced muscle mass, in accordance with the MNA^®^ protocol and evidence from hospitalized adult populations [17]. CC was measured in all participants as part of the full MNA^®^ assessment.

The etiologic component comprised a questionnaire-derived criterion for reduced food intake or impaired assimilation and a protocol-based disease-burden criterion. The questionnaire-derived criterion was considered present when participants reported an intake below 50% of estimated energy requirements for more than one week, any reduction in intake for more than two weeks, or a chronic gastrointestinal condition compromising food digestion or absorption. As individual inflammatory biomarkers were unavailable, disease burden was operationalized a priori as present in all participants based on the underlying ALS diagnosis [2]. Therefore, GLIM classification was based on the presence of at least one phenotypic criterion in addition to the etiologic disease-burden criterion.

Malnutrition severity was graded according to the degree of phenotypic impairment, as recommended by GLIM [15]. Stage 1, moderate malnutrition, was assigned to participants meeting moderate phenotypic thresholds, including weight loss of 5–10% within six months, low BMI, or reduced muscle mass defined as CC < 31 cm. Stage 2, severe malnutrition, was assigned to participants with weight loss exceeding 10% within six months or a severely reduced BMI. When multiple phenotypic criteria were present, the most severe criterion determined the GLIM stage. As no separate threshold for severely reduced muscle mass was applied, CC < 31 cm contributed to Stage 1 classification unless a severe weight-loss or BMI criterion was also present.

#### 2.2.4. Anthropometry and Body Composition

Anthropometric measurements included current and self-reported usual body weight, height, and mid-arm and calf circumferences. Body Mass Index (BMI) was calculated as weight in kilograms divided by height in meters squared. In cases where direct height measurement was not feasible, BMI was estimated from mid-upper arm circumference (MUAC) using the regression equations described by Powell-Tuck and Hennessy [18,19].

Weight change over the six-month period preceding the assessment was recorded and categorized as weight gain, stable weight, moderate weight loss (<10%), or severe weight loss (≥10%). These data, alongside the composite reduced-intake or impaired-assimilation criteria, were utilized for the GLIM diagnostic staging described in the previous section.

Participants were classified using age-specific BMI thresholds to account for age-related variations in body composition. Undernutrition was defined using the World Health Organization (WHO) threshold [20] (<18.5 kg/m^2^) for participants younger than 65 years and the Lipschitz criteria (<22 kg/m^2^) for those aged 65 years or older [21]. The full classification thresholds used in this study are detailed in Table 1.

#### 2.2.5. Social, Environmental, and Healthcare Factors

Social and environmental factors were assessed using indicators of housing adaptations, functional autonomy in activities of daily living (ADLs), use of mobility aids, and availability of transportation. Healthcare access was assessed using the type of healthcare coverage, geographic distance to ALS referral centres, frequency of follow-up consultations, and perceived barriers to accessing ALS-specific services. The intensity of multidisciplinary care was documented by recording the number and specialized types of healthcare professionals providing longitudinal support.

Psychosocial dimensions included living arrangements, familial support structures, and the presence of informal caregivers. Functional dependence was specifically operationalized as the requirement for assistance with ADLs. Self-reported use of complementary or alternative medicine was recorded; however, the specific pattern of use, whether adjunctive to or in place of conventional care, was not assessed.

Perceived psychological stress was evaluated retrospectively for the five-year period preceding the ALS diagnosis. Participants rated their baseline stress levels during this period using a four-point scale (very high, high, low, or very low).

#### 2.2.6. Variable Operationalization and Coding

Prior to analysis, several questionnaire-derived variables were operationalized as binary factors for descriptive and exploratory analyses: caregiver support adequacy, multidisciplinary care, dietary restrictions, communication status, and healthcare accessibility.

The adequacy of caregiver support was operationalized by cross-referencing the requirement for assistance with activities of daily living (ADLs) and its actual availability. Participants were classified as having inadequate caregiver support only if they reported requiring assistance that was not provided; all other participants, including those not requiring help or those receiving necessary aid, were classified as having adequate support (yes/no).

Multidisciplinary care was defined by the number and types of healthcare specialties involved in longitudinal ALS follow-up. Participants receiving care from at least two distinct specialties beyond neurology were classified as receiving multidisciplinary care (yes/no) [9,10].

Dietary restriction was operationalized based on the current feeding modality at assessment. Participants requiring texture-modified diets, exclusive enteral nutrition via gastrostomy, or a combined oral-enteral regimen were classified as having dietary restrictions. Participants maintaining an unrestricted oral diet served as the reference group (yes/no).

Communication status was derived from the presence of communication difficulties and the effectiveness of augmentative and alternative communication (AAC) strategies. For exploratory analysis, this was coded as a binary factor: unresolved or insufficiently compensated communication difficulty (present) versus the absence of difficulty or difficulty effectively compensated by AAC or other strategies (absent).

Healthcare accessibility was operationalized using a five-point scale reflecting the perceived ease of access to ALS follow-up appointments. Responses of “very easy” or “easy” were categorized as accessible healthcare; responses of “neither easy nor difficult,” “difficult,” or “very difficult” were categorized as limited accessibility (yes/no).

### 2.3. Statistical Methodology

The total Mini Nutritional Assessment (MNA^®^) score was analyzed as a continuous measure of broad vulnerability, as it integrates nutritional, anthropometric, functional, and psychosocial domains. To minimize mathematical coupling, variables that directly reproduce MNA^®^ components or serve as close proxies, including BMI, recent weight change, food intake and feeding variables, psychological stress, and mobility assistance, were excluded from the primary predictor set. Nutritional supplementation was also excluded due to the potential for reverse causation and confounding by indication.

The primary analysis utilized a clinically prespecified eight-predictor model, avoiding automated or stepwise selection. The model included recruitment setting (ANOC vs. APELA), age (per 10 years, centered at 65), sex, ALS onset phenotype (bulbar vs. non-bulbar), time since diagnosis (log_2_-transformed and centered at its median to allow interpretation as the effect per doubling of the diagnosis-to-cut-off interval), comorbidity count, education level (≤9 vs. ≥12 years), and respiratory support. Sparse categories were consolidated a priori. With nine regression coefficients, the ratio of observations to coefficients was 10.8.

Coefficients were estimated using ordinary least squares (OLS) with HC3 heteroscedasticity-robust standard errors, t-based 95% confidence intervals, and exact two-sided *p*-values [22]. No data imputation was required, as 100% completion was achieved for all model variables. Internal validation and stability were evaluated using 5000 nonparametric bootstrap resamples.

#### 2.3.1. Model Diagnostics and Robustness

Multicollinearity was assessed using variance inflation factors (VIFs) and the maximum condition index. Influence was assessed using leverage (h), externally studentized residuals, and Cook’s distance (D). Influence flags were prespecified as leverage h > 2p/n, Cook’s D > 4/n, or |externally studentized residual| > 3. All observations were retained in the primary model; observations meeting at least one influence criterion were excluded simultaneously only in the corresponding sensitivity analysis.

#### 2.3.2. Sensitivity Analyses

Prespecified sensitivity analyses included: (i) restriction to participants aged ≥65 years (n=64); (ii) simultaneous exclusion of all observations meeting influence criteria; (iii) Huber robust regression; and (iv) Firth bias-reduced logistic regression for the dichotomized MNA^®^ < 24 outcome. Recruitment-setting-stratified modeling was assessed, but the ANOC subgroup size precluded stable multivariable estimation.

A broader set of candidate variables was explored using elastic-net penalized regression with repeated nested cross-validation and 1000 bootstrap samples to assess selection stability [23]. Because the MNA^®^ < 24 threshold produced quasi-complete separation by recruitment setting (all 20 ANOC participants scored < 24), ordinary logistic regression was inappropriate. Thus, Firth bias-reduced logistic regression with profile penalized-likelihood confidence intervals was employed [24,25]. This analysis was interpreted strictly as a direction-of-association sensitivity check.

#### 2.3.3. Reporting

Adjusted associations are reported as regression coefficients with HC3-robust standard errors and 95% confidence intervals. Overall model performance was summarized using the robust Wald F-test, R^2^, and adjusted R^2^. Bootstrap stability was assessed via sign stability and percentile intervals for the recruitment-setting coefficient. Potential overfitting was quantified using bootstrap optimism-corrected R^2^ and the mean calibration slope.

In line with the cross-sectional design and the exploratory nature of the study, the multivariable model was primarily developed to evaluate adjusted associations with nutritional status rather than for predictive purposes. Consequently, all estimates were interpreted as adjusted associations rather than causal effects. Findings from the elastic-net analysis were treated as hypothesis-generating. All analyses were conducted in Python (version 3.11) using a fixed random seed (20260801) for reproducibility.

### 2.4. Ethical Considerations

All participants were informed of the study’s nature and purpose. Responses were handled confidentially in accordance with applicable data-protection requirements, and written informed consent was obtained. The study was approved by the Hospital Garcia de Orta Ethics Committee on 2 March 2023 and conducted in accordance with the Declaration of Helsinki.

## 3. Results

### 3.1. Sociodemographic Characteristics

**Figure 1:**
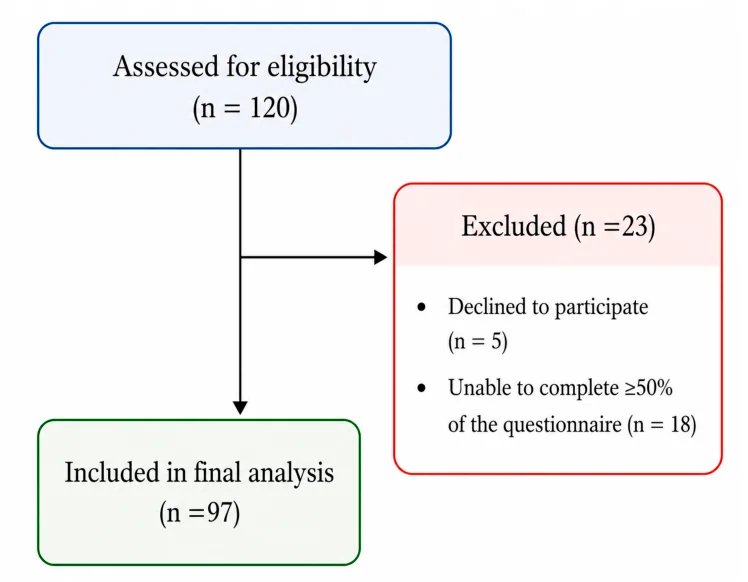
Participant flow diagram illustrating the recruitment process, exclusion criteria, and the final sample size included in the statistical models.

A participant flow diagram (Figure 1) summarizes screening, exclusions, and the final analytical sample of 97 participants: 77 recruited through APELA and 20 through ANOC.

The mean age of the cohort was 66.2 years (SD = 10.5), ranging from 28 to 91 years; 33 participants (34.0%) were younger than 65 years, while 64 (66.0%) were 65 years or older. The majority of participants were male (55.7%). Educational attainment was diverse: 4.1% had no formal education, 21.6% completed primary education, 15.5% completed lower secondary, and 28.9% completed upper secondary education. Additionally, 23.7% held a bachelor’s degree, and 6.2% held a master’s or doctoral degree. For the multivariable modeling, these levels were consolidated into participants with ≤9 years (41.2%) versus ≥12 years (58.8%) of formal education to ensure model parsimony.

Regarding employment status, the majority were retired (75.3%), while 6.2% were actively employed, 3.1% were unemployed, and 15.5% reported other statuses, including domestic work or medical leave. Geographically, participants were distributed across mainland Portugal, with the majority residing in the southern coastal region (48.5%), followed by the northern coastal region (23.7%) and the central inland region (21.6%). Smaller proportions were identified in the central coastal (5.2%) and southern inland (1.0%) regions.

### 3.2. Clinical Features

Regarding ALS onset phenotype, 40.2% of participants (*n =* 39) presented with bulbar-onset disease, 49.5% with spinal-onset, and 10.3% were classified as unclassified or other forms. For multivariable modeling, bulbar onset was compared with a non-bulbar category (59.8%) that combined spinal and other phenotypes to ensure statistical robustness. The median time since diagnosis was 702.0 days (IQR = 345.0–1250.0).

Eating-related difficulties were highly prevalent, with 66.0% of participants reporting general eating problems and 50.5% reporting specific dysphagia for liquids. These clinical challenges were reflected in feeding modalities: 37.1% maintained an unrestricted oral diet, 30.9% required texture-modified diets, and 32.0% relied on enteral nutrition (21.6% exclusive PEG and 10.3% a combined oral-enteral regimen). Use of oral nutritional supplements (ONS) was reported by 56.7% of participants, and more than half of the cohort (54.6%) required respiratory support, reflecting the progressive nature of the disease and its impact on bulbar and respiratory functions.

Communication status, a key indicator of clinical severity in this cohort, was categorized into three groups: 39.2% of participants (*n =* 38) reported no communication difficulty; 10.3% (*n =* 10) had difficulties that were effectively compensated through augmentative and alternative communication (AAC) systems; and 50.5% (*n =* 49) reported unresolved or insufficiently compensated communication difficulty. This latter group represented a significant portion of the sample, potentially reflecting a multidimensional barrier to maintaining adequate nutritional and social support.

### 3.3. Nutritional Status

The nutritional profile of the cohort was multidimensionally assessed using the Mini Nutritional Assessment (MNA^®^), Body Mass Index (BMI), and the Global Leadership Initiative on Malnutrition (GLIM) criteria. The mean MNA^®^ score for the total sample was 19.69 (SD = 4.53), positioning the cohort average within the at-risk range (17.0–23.5 points). Consistent with this, the mean BMI was 22.7 kg/m^2^ (SD = 4.6), with values ranging from 14.3 to 31.8 kg/m^2^.

Under the study-specific GLIM operationalization, 42.3% of participants (*n* = 41) were classified as moderately malnourished, 43.3% (*n =* 42) as severely malnourished, and 14.4% (*n =* 14) as not malnourished. These findings underscore the high nutritional vulnerability of this ALS population, even when clinical indicators such as stable weight over the previous six months were reported by 48.5% of the participants. In contrast, clinically significant weight loss was prevalent, with 21.6% and 22.7% of the cohort experiencing moderate (<10%) and severe (≥10%) weight loss, respectively, while only 7.2% reported weight gain.

Anthropometric data further detailed the participants’ physical status, with a mean body weight of 62.7 kg and a mean height of 1.65 m. The biological impact of the disease was further evidenced by the composite reduced-intake or impaired-assimilation criterion, which was positive in 38.1% of the participants, reflecting the significant barriers to maintaining adequate nutritional intake in this clinical setting.

### 3.4. Social and Environmental Needs

Regarding the home environment, 63.7% of participants lived in homes with some level of adaptation, 26.5% lived in fully adapted homes, and 9.8% lived in non-adapted homes. Reported unmet needs included bathroom adaptations, stair lifts, and transfer aids. Assistance with activities of daily living (ADLs) was required by 74.2% of the cohort (*n =* 72).

The availability of this assistance was high; based on the study-specific coding defined in Section 2.2.6, only 2.1% of participants (*n =* 2) were classified as having inadequate caregiver support (requiring assistance but not receiving it), while 72.2% (*n =* 70) reported having the necessary assistance. Regarding mobility, 70.1% of participants used walking aids, whereas 29.9% did not require them at the time of assessment. Mobility-related environmental support was reported by 31.7% of the cohort.

The most frequently reported mode of transportation was a car driven by another person (32.0%), followed by ambulance transport (28.9%) and self-driving without vehicle adaptations (18.5%). Use of taxis or ride-hailing services (14.4%) and public transportation (4.1%) was less frequent. Only 2.1% of participants drove adapted vehicles.

### 3.5. Healthcare Access, Support, and Psychosocial Needs

Analysis of healthcare coverage revealed that 58.8% of participants were covered exclusively by the National Health Service (SNS), while 21.6% reported private health insurance and 19.6% had coverage through the public assistance system (ADSE). Regarding living arrangements, 90.7% of the cohort resided with family members, 7.2% lived alone, and 2.1% were in care institutions.

Geographic accessibility to specialized ALS centers varied, with distances ranging from 3 to 300 km (Mean = 35 km; Median = 15 km). The frequency of follow-up consultations was predominantly every six months (57.7%), followed by every three months (36.1%) and monthly (5.2%); irregular follow-up was reported by only 1.0% of participants. Perceived ease of access to care was reported by 51.5% of the cohort, while 20.7% reported significant difficulties. Multidisciplinary care was widely implemented, with 61.8% of participants receiving support from more than five clinical specialties. While neurologist follow-up was nearly universal (99.0%), other frequently involved professionals included physical therapists and dietitians (81.4%), speech-language therapists (59.8%), nurses (48.5%), and social workers (40.2%). The use of complementary or alternative therapies was reported by 16.5% of participants.

Regarding pre-diagnostic psychosocial factors, 51.5% of the cohort retrospectively reported high or very high levels of perceived psychological stress during the five years preceding their ALS diagnosis, while 48.5% reported low or very low stress levels during the same period.

### 3.6. Comparison of Clinical and Nutritional Profiles Across ALS Care Settings

Marked differences in clinical and nutritional characteristics were observed between the recruitment sources. Participants recruited at ANOC (*n* = 20) presented a more advanced clinical profile, with a substantially longer median time since diagnosis than that of the APELA cohort (1232.5 vs. 610.0 days; *n* = 77). While the mean age and body weight were relatively similar between groups (ANOC: 67.0 years and 60.2 kg; APELA: 66.2 years and 63.3 kg), the distribution of onset phenotypes differed markedly. Bulbar-onset disease was the predominant phenotype in the ANOC group (75.0%), whereas the spinal-onset phenotype was more common in the APELA group (57.1%). Consistent with the prespecified modeling strategy, non-bulbar phenotypes were aggregated for subsequent multivariable analysis.

Nutritional vulnerability was also more pronounced in the hospital-based outpatient clinic. The mean MNA^®^ score was 16.98 (SD = 4.06) in ANOC, compared with 20.39 (SD = 4.40) in APELA; notably, all ANOC participants scored below the 24-point threshold for normal nutritional status, whereas 29.9% (*n* = 23) of APELA participants were classified within the normal range. Under the study-specific GLIM framework, severe malnutrition was identified in 50.0% of the ANOC group and 41.6% of the APELA group. Weight-gain reports were infrequent across both settings (ANOC: 10.0%; APELA: 6.5%). Mean BMI was lower in the ANOC group (21.4 vs. 23.0 kg/m^2^), further reflecting the case-mix differences that necessitated the inclusion of recruitment setting as a mandatory covariate in the multivariable model.

### 3.7. Multivariable Linear Model for MNA^®^ Score

The primary model included all 97 participants, with complete data for all prespecified predictors.

In the adjusted primary model, recruitment at ANOC was associated with a 4.48-point lower total MNA^®^ score than APELA (Coefficient = −4.477; HC3 SE = 1.305; 95% CI −7.071 to −1.884; exact *p* = 0.0009). Bootstrap sign stability for this estimate reached 99.98%, with a percentile interval of −6.768 to −1.848 points, indicating high directional stability of this association within the sample. Estimates for age, sex, onset phenotype, time since diagnosis, comorbidity burden, education, and respiratory support were imprecise, with 95% confidence intervals spanning the null value; these results suggest uncertainty rather than evidence of no association. Detailed model parameters are provided in Table 2.

**Figure 2:**
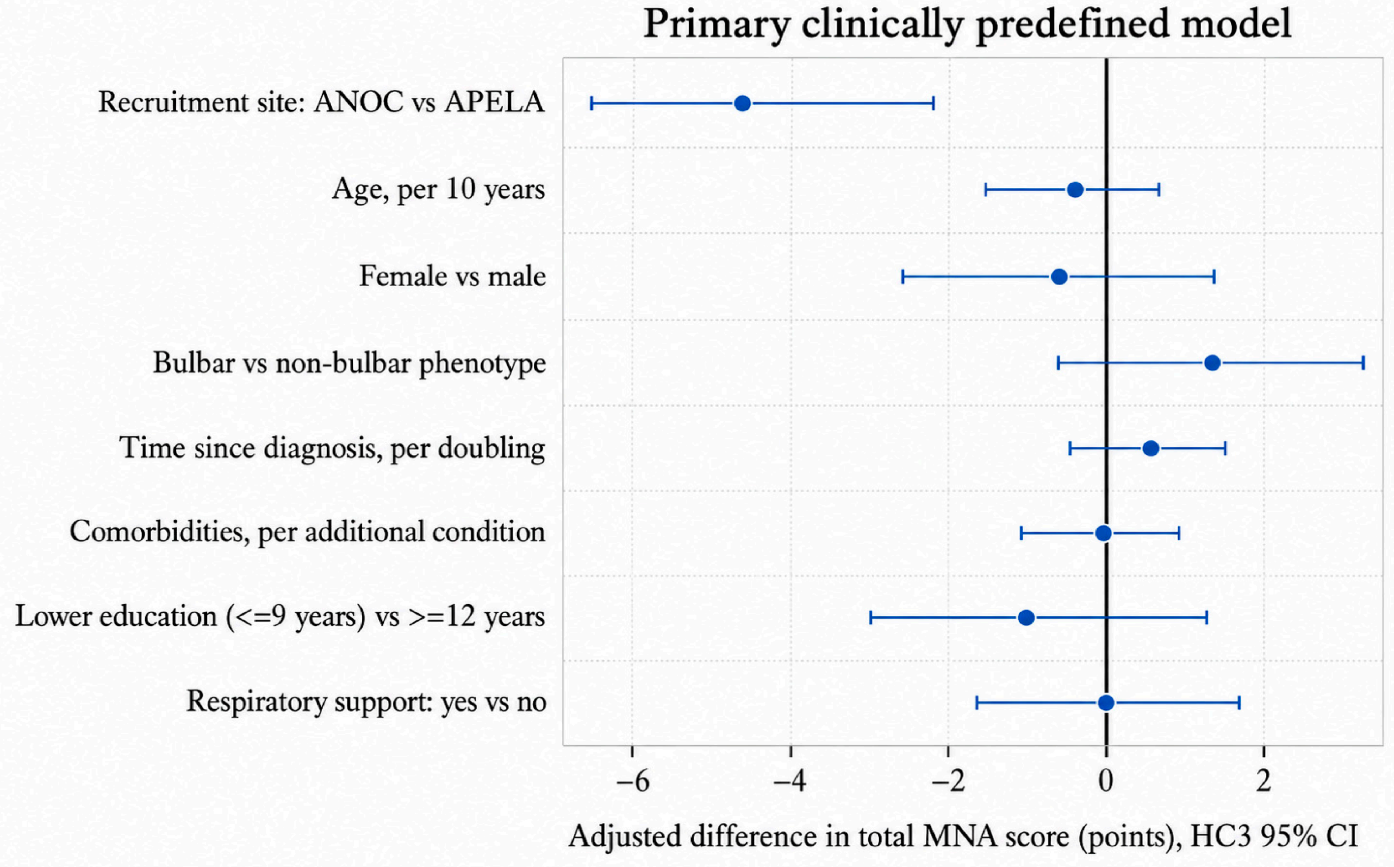
Adjusted mean differences in total MNA^®^ points for the predictors with HC3 95% confidence intervals. The recruitment setting (ANOC vs. APELA) was clearly associated with the total MNA^®^ score, whereas the remaining clinical and sociodemographic estimates were inconclusive, with 95% confidence intervals spanning the null value.

The overall model was statistically significant (Robust Wald F(8,88) = 2.175; *p* = 0.037), explaining 14.5% of the variance (Adjusted R^2^= 0.067). However, internal validation via bootstrap optimism correction yielded an R^2^ of −0.005 and a calibration slope of 0.698 (95% CI 0.439–1.028). While this negative corrected R^2^ indicates that the model lacks the stability required for individual-level prediction or prospective forecasting, the consistency of the recruitment-setting coefficient across sensitivity analyses supports its stability as an adjusted association within this cohort (Figure 2).

No observations were removed from the primary analysis. Four observations met at least one prespecified influence criterion (leverage h > 2p/n, Cook’s D > 4/n, or |externally studentized residual| > 3); however, none exhibited an externally studentized residual exceeding 3, and the maximum leverage was 0.191675. In a sensitivity analysis excluding these four observations simultaneously, the ANOC coefficient remained stable (Coefficient = −3.746; 95% CI −5.943 to −1.549; *p* = 0.0011). Huber robust regression produced a closely similar estimate of −4.610 points. Among participants aged ≥65 years (*n* = 64), the ANOC coefficient remained negative, albeit with lower precision (−3.632; 95% CI −7.308 to 0.044; *p* = 0.0527). Collinearity was not a concern, with variance inflation factors ranging from 1.034 to 1.478 and a maximum condition index of 2.061.

In the exploratory elastic-net analysis applied to a broader, non-overlapping candidate set, unresolved communication difficulty (selected in 100% of bootstrap samples), recruitment setting (99.2%), bulbar phenotype (94.4%), and frequent ALS follow-up (93.5%) were identified as the most stable markers. These findings are exploratory and may reflect underlying clinical severity, referral pathways, or surveillance intensity; they should not be interpreted as independently proven causal predictors. Finally, as dichotomization at MNA^®^ < 24 produced quasi-complete separation by recruitment setting, Firth bias-reduced logistic regression was performed as a sensitivity analysis. The ANOC odds ratio for MNA^®^ < 24 was 34.6, but the 95% confidence interval was wide (3.38–4789.42), reflecting site-related separation rather than a precise estimate of the effect size (*p* = 0.0008). This analysis supports the direction of the primary continuous-outcome result and serves as a secondary sensitivity check.

## 4. Discussion

This cross-sectional study characterized the clinical, nutritional, social, and healthcare-related features of 97 adults with ALS recruited through APELA and ANOC in Portugal. The prespecified multivariable analysis evaluated adjusted associations with the total MNA^®^ score, which was interpreted as a broad measure of nutritional and functional vulnerability. Recruitment setting was the only prespecified factor showing a clear adjusted association with total MNA^®^ score, whereas the estimates for age, sex, ALS onset phenotype, time since diagnosis, comorbidity burden, educational level, and respiratory support were imprecise, with confidence intervals that included the null value. Participants recruited through ANOC had an adjusted MNA^®^ score that was 4.48 points lower than those recruited through APELA (Coefficient = −4.477; HC3 95% CI −7.071 to −1.884; *p* = 0.0009). This association remained directionally consistent after exclusion of influential observations and in robust regression analyses, although it was less precise among participants aged 65 years or older. Because the two recruitment settings differed substantially in referral pathways and clinical case mix, including time since diagnosis, ALS onset phenotype, and unadjusted MNA^®^ score, this finding should not be interpreted as an effect of the care delivered at either setting. Rather, the recruitment setting is likely to represent differences in referral patterns, clinical characteristics, and unmeasured factors between the two study populations. The model showed modest apparent explanatory performance and insufficient internal validation for individual-level prediction; accordingly, its findings should be interpreted as adjusted associations within this sample rather than as a prediction model or causal estimates.

### 4.1. Sociodemographic and Clinical Dimensions

The mean age of participants was 66.2 years, and men were slightly more represented than women, consistent with demographic and epidemiological characteristics commonly reported in ALS cohorts [2]. Educational attainment varied considerably, with a substantial proportion of participants having completed only basic education. Although education is not equivalent to socioeconomic position, it may reflect broader differences in health literacy, access to health information, and the ability to navigate complex healthcare pathways. Education and occupation have been investigated as epidemiological correlates of ALS [26], and population-based evidence suggests that socioeconomic indicators are associated with ALS mortality patterns [27]. However, detailed socioeconomic information, such as income, occupation, household deprivation, and material resources, was not collected in the present study. Educational attainment should therefore be interpreted as one component of the participants’ social context rather than as a comprehensive measure of socioeconomic status. Future studies should incorporate multidimensional socioeconomic indicators to better characterize the social and structural determinants potentially relevant to nutritional vulnerability in ALS.

Nearly half of the cohort presented with spinal-onset ALS, whereas 40.2% presented with bulbar-onset disease. Bulbar onset was more frequent among participants recruited at ANOC than among those recruited through APELA, occurring in 75.0% and 31.2% of participants, respectively. This difference reflects the distinct clinical and referral profiles of the two recruitment settings. Bulbar involvement is clinically relevant in ALS because swallowing and communication difficulties are common and may complicate feeding, nutritional counseling, and interactions with healthcare professionals. The predominance of bulbar-onset disease among participants recruited at ANOC is compatible with the referral profile of this setting, which included individuals receiving specialised nutritional and supportive care.

Nevertheless, these differences should be viewed as characteristics of the populations recruited at each setting rather than as evidence that the recruitment setting itself determines clinical or nutritional status. More than 60% of participants reported communication difficulties; however, only a minority reported effective compensation using augmentative and alternative communication strategies. This finding indicates that communication impairment and the availability of compensatory strategies were important aspects of the clinical profile of this cohort. Notably, unresolved communication difficulty was the most consistently selected marker in the exploratory elastic-net analysis (bootstrap selection frequency 100%), suggesting that it may warrant further investigation as a potential correlate of nutritional vulnerability in future prospective studies. However, given the exploratory nature of this analysis and the risk of reverse causation, this finding should not be interpreted as evidence of an independent causal association. Communication difficulties may make it more difficult for individuals with ALS to report symptoms, express eating-related concerns, participate in nutritional decision-making, and maintain effective interactions with caregivers and healthcare professionals. They may also increase the complexity of delivering individualized dietary counseling and coordinating multidisciplinary care.

From a clinical perspective, these findings support integrating routine speech and language assessments into multidisciplinary ALS care, along with timely access to augmentative and alternative communication strategies when indicated. These approaches may facilitate symptom reporting, communication with caregivers and healthcare professionals, and participation in decisions about nutritional and supportive care. Prospective studies are needed to determine whether earlier implementation of these strategies improves communication, nutritional monitoring, or other patient-centered outcomes.

### 4.2. Nutritional Risk and Functional Decline

Nutritional vulnerability was common in this cohort. The mean MNA^®^ score was 19.69 (SD = 4.53), within the range conventionally interpreted as nutritional risk. Under the prespecified study-specific GLIM operationalization, 42.3% of participants met criteria for moderate malnutrition and 43.3% for severe malnutrition, while 14.4% did not meet criteria for malnutrition. Because the disease-burden criterion was applied uniformly and reduced muscle mass was assessed using calf circumference rather than instrumental body-composition measurements, these prevalence estimates should be interpreted in light of those operational decisions. The mean BMI of 22.7 kg/m^2^ did not capture the weight-loss and muscle-mass components reflected in the broader assessments, illustrating the limitations of interpreting BMI in isolation [8,28].

Weight loss was common: 44.3% of participants were classified as having moderate or severe weight loss over the preceding six months, and more than one-third met the composite criterion for reduced intake or impaired assimilation. These findings align with previous studies linking dysphagia, hypermetabolism, and functional decline to nutritional deterioration in ALS [28,29]. Nutritional status has prognostic value in ALS, with lower nutritional indices associated with shorter survival in cohort studies [3]. Although survival outcomes were not assessed here, the observed burden of nutritional impairment supports careful nutritional monitoring but does not permit prognostic conclusions for individual participants.

Although more than half of participants reported using oral nutritional supplements (ONS), malnutrition remained widespread. This cross-sectional observation does not imply that ONS use is associated with poorer nutritional status. The pattern is plausibly explained by confounding by indication, as ONS are commonly initiated after weight loss, reduced oral intake, or other evidence of nutritional deterioration [28,30]. The persistence of malnutrition among ONS users may therefore reflect the severity or timing of the nutritional compromise that prompted supplementation rather than an adverse effect of supplementation [30,31]. Prospective studies are needed to clarify whether the timing and adequacy of nutritional intervention influence nutritional trajectories in ALS [8,29].

### 4.3. Social and Environmental Vulnerabilities

Beyond biomedical factors, the cohort exhibited significant environmental vulnerability. Only one-quarter of participants resided in fully adapted homes, while the majority occupied dwellings requiring further modifications to ensure safety and autonomy. Structural barriers, such as inaccessible bathrooms and the lack of stair lifts or transfer aids, were prevalent and may exacerbate the functional limitations imposed by ALS. Although more than 70% of the cohort required assistance with activities of daily living (ADLs), the study-specific clinical coding revealed that nearly all participants receiving help (72.2%) reported having adequate caregiver support. Only a marginal proportion (2.1%) fell into the high-vulnerability category of requiring assistance without having it available, suggesting that, in this sample, environmental barriers and functional dependency outweigh the immediate lack of human support.

Transportation limitations further compounded these environmental challenges. Despite the high frequency of mobility aid use (70.1%), access to adapted private vehicles and specialized public transportation remained extremely limited. This lack of independent or adapted mobility solutions may increase social isolation and create significant hurdles in accessing specialized ALS centers, particularly for those residing at greater distances. These findings suggest that for individuals with ALS in Portugal, the primary social and environmental vulnerabilities are rooted in the physical barriers of the home and the lack of structural transportation support, rather than a deficit in informal caregiving resources.

### 4.4. Healthcare Access and Multidisciplinary Follow-Up

Most participants were followed by multiple healthcare professionals, underscoring the recognized importance of multidisciplinary care in ALS. Nearly all had neurologist follow-up, and many were supported by dietitians, speech-language therapists, nurses, and other specialists. However, the prevalence of malnutrition and unmet social needs suggests that multidisciplinary involvement alone may be insufficient to address the complex, multidimensional needs of this population.

In the exploratory elastic-net analysis, ALS follow-up at least every three months was among the most consistently selected markers (bootstrap selection frequency 93.5%) and was associated with a lower total MNA^®^ score in the penalized model. However, this finding is exploratory and should not be interpreted as a causal effect of follow-up frequency on nutritional status. More frequent follow-up may reflect greater clinical complexity, nutritional vulnerability, surveillance intensity, referral patterns, or service organization. Furthermore, follow-up frequency was not included in the prespecified primary model, and its selection in the elastic-net analysis reflects a broader candidate set examined under penalized regression rather than a confirmatory finding. Prospective longitudinal studies are needed to determine the independent relationship between follow-up patterns and nutritional outcomes.

The use of complementary or alternative medicine by 16.5% of participants may reflect unmet needs, perceived gaps in conventional care, or a search for personal agency in the context of a progressive disease. More than half of participants retrospectively reported high or very high stress during the five years preceding ALS diagnosis. This interval was intended to capture experiences around symptom onset, diagnostic delay, and uncertainty, as documented in qualitative accounts of the ALS diagnostic journey [32]. However, retrospective recall is vulnerable to bias, and the item cannot distinguish stress preceding symptom onset from stress arising during the diagnostic process. Prospective research is needed to examine psychological experiences before and after diagnosis in relation to nutritional status and disease progression.

### 4.5. Clinical and Nutritional Heterogeneity Between Care Settings

Participants recruited through ANOC showed a more clinically vulnerable profile than those recruited through APELA, including more frequent bulbar onset, longer time since diagnosis, and lower BMI and MNA^®^ scores. These contrasts are clinically relevant in a disease in which nutritional status and specialized nutritional support are closely linked to disease burden and outcomes [33,34], but in the present study they primarily indicate heterogeneity in case mix and referral pathways. Recruitment setting may therefore capture differences in disease severity, nutritional needs, referral timing, access to specialized interventions, and other unmeasured clinical or social characteristics.

The study was not designed to compare the effectiveness of APELA and ANOC care. Participants were not assigned to settings, standardized ALS severity measures were unavailable, and nutritional status was assessed at a single time point. Accordingly, the site-related difference should be interpreted as a characteristic of the populations entering each care pathway rather than as evidence that one setting produces better or worse nutritional outcomes. Evidence from ALS centres indicates that specialist and multidisciplinary care can influence resource use and survival [35,36], but whether coordinated community and hospital pathways improve nutritional trajectories requires prospective evaluation.

### 4.6. Interpretation of the Prespecified Multivariable Model

The primary model treated the total MNA^®^ score as an integrative measure of nutritional and functional vulnerability rather than as a purely metabolic outcome. Because several candidate variables directly reproduce MNA^®^ components, overlapping measures were excluded a priori to reduce mathematical coupling. This interpretation is consistent with recent morphofunctional work applying GLIM-related assessment in ALS [37].

Recruitment setting was the only prespecified covariate with a clear adjusted association with total MNA^®^ score, and its direction was consistent across bootstrap and sensitivity analyses. In contrast, estimates for age, sex, bulbar phenotype, time since diagnosis, comorbidity burden, education, and respiratory support were imprecise. These results do not establish absence of association for those variables; rather, they indicate limited precision after multivariable adjustment. Education, in particular, is only a partial proxy for socioeconomic context and health literacy [38].

The model’s modest explanatory performance and poor optimism-corrected predictive performance reinforce that it should be interpreted as an exploratory association model, not as a clinical prediction or risk-stratification tool. The elastic-net analysis likewise remains hypothesis-generating: the recurrent selection of communication difficulty, recruitment setting, bulbar phenotype, and follow-up frequency may reflect disease severity, referral patterns, surveillance intensity, or reverse causation. Overall, the findings support a multidimensional interpretation of nutritional vulnerability while emphasizing the need for longitudinal studies that include more comprehensive measures of ALS severity and social context.

### 4.7. Limitations and Future Directions

Several limitations should be considered. The cross-sectional design limits temporal and causal interpretation, and the modest sample size reduced the precision of some estimates, particularly in subgroup and sensitivity analyses. The small ANOC subgroup (*n* = 20) also precluded stable multivariable estimation within the setting.

Residual confounding by disease severity remains possible because standardized ALS-specific functional assessment (ALSFRS-R) [39], objective respiratory-function measures, symptom-onset-based disease duration, and neurofilament light chain [40] were unavailable. Time since diagnosis was therefore used as a pragmatic indicator of disease course. Questionnaire-derived measures of functional dependency were not included in the primary model because of their conceptual overlap with components of the total MNA^®^ score.

Several variables, including dietary intake and pre-diagnostic psychological stress, relied on participant or caregiver report and were therefore susceptible to recall and measurement error. In addition, 18 screened individuals were excluded because they were unable to complete at least 50% of the questionnaire, potentially underrepresenting participants with greater communication impairment or clinical vulnerability.

Nutritional assessment also requires caution. The use of the MNA^®^ in the 33 participants aged <65 years was exploratory because equivalent validity has not been established in younger adults with ALS. GLIM classification relied on a study-specific operationalization in which ALS fulfilled the disease-burden criterion and calf circumference < 31 cm was used as a pragmatic proxy for reduced muscle mass; in ALS, neurogenic muscle atrophy may therefore contribute to overestimation of malnutrition prevalence. BMI, weight change, and GLIM classification were not evaluated as alternative multivariable outcomes, limiting assessment of robustness across nutritional endpoints. Future longitudinal studies should incorporate repeated nutritional assessments together with ALSFRS-R, objective respiratory and body-composition measures, and standardized psychosocial and social indicators.

## 5. Conclusions

In this cross-sectional study of 97 adults with ALS, nutritional and functional vulnerability was highly prevalent. The mean MNA^®^ score fell within the nutritional risk range, and 85.6% of participants met study-specific GLIM criteria for moderate or severe malnutrition. These findings underscore the need for a multidimensional approach to nutritional assessment, as BMI alone proved insufficient to capture the phenotypic and functional complexity identified by the MNA^®^ and GLIM frameworks.

In the prespecified adjusted model, recruitment setting was the most consistent correlate of nutritional status, with participants recruited via ANOC having lower MNA^®^ scores than those from APELA. This association likely reflects systematic differences in clinical case mix, referral pathways, and disease severity between the two populations, rather than a causal effect of the care setting itself. Complementary insights from the MNA^®^ and GLIM criteria highlight that malnutrition in ALS is a complex construct shaped by disease-related impairment, social context, and access to healthcare.

These results are hypothesis-generating and underscore the need for regular, comprehensive nutritional screening that extends beyond weight monitoring. Future longitudinal research, incorporating standardized measures of functional status (ALSFRS-R), objective body composition, and more detailed indicators of socio-environmental support, is essential to clarify the temporal drivers of nutritional decline and to optimize multidisciplinary care strategies for this vulnerable population.

## Figures and Tables

**Table 1:** Body mass index (BMI) classification thresholds by age group.

	Low	Normal	High
<65 Years	<18.5 kg/m^2^	≥18.5 & <25 kg/m^2^	≥25 kg/m^2^
≥65 Years	<22 kg/m^2^	≥22 & <27 kg/m^2^	≥27 kg/m^2^

**Table 2:** Multivariable linear regression analysis of factors associated with total MNA^®^ score in participants with ALS (*N =* 97).

Predictor	Estimate	HC3 SE	95% CI	T	df	*p* Value
Intercept (APELA, male, age 65, non-bulbar, higher education, no respiratory support)	20.867	1.131	18.619 to 23.115	18.446	88	<0.0001
Recruitment site: ANOC vs. APELA	−4.477	1.305	−7.071 to −1.884	−3.431	88	0.000918
Age, per 10 years	−0.401	0.539	−1.473 to 0.670	−0.744	88	0.458587
Female vs. male	−0.602	0.951	−2.492 to 1.289	−0.632	88	0.528768
Bulbar vs. non-bulbar phenotype	1.272	1.035	−0.785 to 3.329	1.229	88	0.222460
Time since diagnosis, per doubling	0.543	0.426	−0.304 to 1.390	1.275	88	0.205646
Comorbidities, per additional condition	−0.110	0.391	−0.886 to 0.667	−0.281	88	0.779717
Lower education (≤9 years) vs. ≥12 years	−0.620	1.008	−2.624 to 1.384	−0.615	88	0.540006
Respiratory support: yes vs. no	−0.041	0.934	−1.896 to 1.815	−0.043	88	0.965405

Legend: HC3 SE, heteroskedasticity-consistent standard error; CI, confidence interval; MNA, Mini Nutritional Assessment.

## Data Availability

The data are not publicly available because they contain clinical information subject to ethics and data-governance restrictions. De-identified data may be made available by the corresponding author upon reasonable request and subject to the required authorization.

## Abbreviations

The following abbreviations are used in this manuscript:

ADLs	Activities of Daily Living
ADSE	Portuguese Public Health Assistance for Civil Servants
ALS	Amyotrophic Lateral Sclerosis
ALSFRS-R	Amyotrophic Lateral Sclerosis Functional Rating Scale-Revised
ANOC	Artificial Nutrition Outpatient Consultation
APELA	Portuguese Association of Amyotrophic Lateral Sclerosis
BMI	Body Mass Index
ESPEN	European Society for Clinical Nutrition and Metabolism
GLIM	Global Leadership Initiative on Malnutrition
MNA^®^	Mini Nutritional Assessment
MUAC	Mid-Upper Arm Circumference
ONS	Oral Nutritional Supplements
PEG	Percutaneous Endoscopic Gastrostomy
SNS	Serviço Nacional de Saúde

## Appendix A. Nutritional Status Assessment and Societal Needs in People with Amyotrophic Lateral Sclerosis (ALS)

**Patient Origin**

**1. Where is the patient being seen?**

□ APELA appointment

□ ANOC appointment (artificial nutrition clinic)

**Sociodemographic Characterisation**

**2. Sex**

□ Male

□ Female

**3. Highest level of education completed**

□ No formal schooling

□ 4th grade

□ 9th grade (or equivalent)

□ 12th grade (or equivalent)

□ Bachelor’s degree (or equivalent)

□ Master’s or Doctoral degree

**4. Year of birth**

______________________________

**5. Municipality of residence**

______________________________

**6. Weight 6 months ago (kg)**

______________________________

**7. Current weight (kg)**

______________________________

**8. Height (m)**

______________________________

**9. BMI (kg/m^2^)**

______________________________

**10. What is your current work status?**

□ Employed

□ Student

□ Unemployed

□ Retired

□ Temporary work disability (medical leave)

□ Homemaker (household tasks)

**11. What is your health coverage? (Select all that apply.)**

□ National Health Service

□ Sub-system (e.g., ADSE, ADM, IASFA, …)

□ Health insurance

□ Private health plan

□ Other (please specify)

**12. In what month and year were you diagnosed with ALS?**

(mm/yyyy)

____/____

**13. What was the initial presentation of your disease?**

□ Unknown

□ Spinal

□ Bulbar

□ Respiratory

□ Axial

□ Diffuse

**14. How would you rate your level of psychological stress on most days, in the 5 years prior to your ALS diagnosis?**

□ Very high

□ High

□ Low

□ Very low

**15. Where do you get information about the disease and related topics? (Select all that apply).**

□ Patient association (APELA)

□ Physician

□ Nurse

□ Internet

□ International patient associations

□ Family and friends

□ Other (please specify)

**16. You currently live:**

□ Alone, at home

□ With family, at home

□ In an institution (nursing home, long-term care unit, or similar)

□ Hospitalised

□ Other (please specify)

**I. Needs Assessment Based on the Patient’s CURRENT Situation**

**17. Have you made changes to your home because of ALS?**

□ Yes

□ No, because I could not

□ No, because I did not need to

**18. If you made changes at home, please indicate which ones:**

______________________________

**19. How would you rate how well your home is adapted to your needs?**

□ Fully adapted

□ Partially adapted

□ Poorly adapted

□ Not adapted at all

**20. What do you consider to be the main unmet needs in your home (what you do not have and miss most)?**

______________________________

**21. Do you need help with day-to-day tasks at home (such as dressing, meal preparation, or personal hygiene)?**

□ Yes, and I have that help

□ Yes, but I do not have that help

□ No

**22. Do you use any of the following walking aids? (Select all that apply.)**

□ None

□ Cane/crutch

□ Walker

□ Manual wheelchair

□ Powered wheelchair

□ Other (please specify)

**23. When you leave home, most of the time you do so:**

□ Alone

□ Accompanied

□ Often I do not leave home because I do not have anyone to accompany me

□ I avoid leaving home because I do not feel comfortable

**24. Do you need help to transfer, for example from bed to chair, or from chair to sofa?**

□ No

□ Yes

□ Yes, but I do not have help

**25. Do you have difficulty swallowing, or saliva problems, that affect how you eat or drink liquids?**

□ Yes

□ No

**26. How do you drink liquids? (Select all that apply.)**

□ Regular water

□ With thickener

□ Gelatin

□ Thickened/gelled water

□ Other (please specify)

**27. Have you reduced food intake to less than 50% of energy needs for more than 1 week, OR any reduction for more than 2 weeks, OR do you have a chronic GI condition that compromises absorption or digestion of food?**

□ Yes

□ No

□ Other (please specify)

**28. How do you eat? (Select all that apply.)**

□ Regular diet

□ Soft diet

□ Oral formulas

□ PEG (Percutaneous Endoscopic Gastrostomy)

□ Nasogastric tube

□ Other (please specify)

**29. If you have a PEG, when was it placed?**

(mm/yyyy)

____/____

**30. In addition to your regular diet, do you take oral nutritional supplements (ONS)?**

□ Yes

□ No, because they were not recommended

□ No, because they are expensive

□ No, because I am fed through PEG

**31. If you take ONS, on average, how many do you have per day?**

□ <1 per day (not every day)

□ 1 to 2

□ 3 or more

**32. At what time of day do you take ONS? (Select all that apply).**

□ Breakfast

□ Mid-morning

□ Lunch

□ Mid-afternoon

□ Dinner

□ Bedtime snack

**33. Do you replace LUNCH or DINNER with ONS?**

□ Yes

□ No

**34. Do you use any equipment to assist your breathing?**

□ Yes

□ No

**35. If YES, what ventilatory support equipment do you use? (Select all that apply).**

□ Non-invasive ventilation (NIV) (nasal mask and a small portable ventilator)

□ Nasal positive pressure ventilation (NPPV)

□ Oral positive pressure ventilation

□ BiPAP-S/T ventilatory support system

□ Tracheostomy

□ Need for oxygen

**36. If YES, who monitors your ventilatory support?**

______________________________

**Medication, Comorbidities, and Follow-up**

**37. Who picks up your medications from the pharmacy?**

□ Me (the patient)

□ Informal caregiver

□ The pharmacy delivers to my home

□ I order online

□ Other (please specify who)

**38. In addition to riluzole, do you take any other medication(s) regularly?**

□ Yes

□ No

□ I do not take riluzole

**39. If you answered “Yes,” please indicate which medications and for what purpose:**

______________________________

**40. Do you take any vitamin supplement (except ONS), or natural products regularly?**

□ Yes

□ No

**41. If you answered “Yes,” please indicate which and for what purpose:**

______________________________

**42. Do you have any of the following comorbidities? (Select all that apply).**

□ Hypertension (high blood pressure)

□ Diabetes

□ Hypercholesterolemia (high cholesterol)

□ Chronic heart disease (e.g., heart failure, ischemic heart disease)

□ Chronic respiratory disease (e.g., asthma, COPD)

□ Depression

□ Anxiety

□ Other (specify)

**43. This year, did you get the flu vaccine?**

□ Yes

□ No

**44. Have you ever received the pneumonia vaccine?**

□ Yes

□ No

□ Other

**45. Are you vaccinated against COVID-19?**

□ No, I did not get vaccinated

□ Yes

**46. Do you have difficulties communicating (being understood)?**

□ No

□ Yes

□ Yes, but they are resolved

**47. If you answered “Yes, but they are resolved” in the previous question, what do you use? (Please indicate which devices/equipment you use to communicate.) (Select all that apply).**

□ Tablet, cellphone, computer, or similar

□ Notebook/notepad (handwriting)

□ GRID or similar application

□ Other (which?)

**48. When you have an exacerbation/worsening of your disease, who do you contact? (Select all that apply).**

□ Neurologist

□ Primary care/Family medicine physician

□ Physical therapist

□ Nurse

□ Speech-language therapist

□ Other (specify)

**49. Where are you followed for ALS? (You may indicate more than one place; please list the main one first.) (Select all that apply).**

□ APELA

□ Public hospital

□ Private hospital

□ Health center

□ Private office/clinic

□ Other (which)

**50. Approximate distance (km) from your home to the location where you are followed for ALS**

______________________________

**51. On average, how long (in hours) does it take from your home to where you are followed for ALS? (If less than 1 h, mark 0).**

______________________________

**52. How do you travel to where you are followed for ALS? (Select all that apply).**

□ I drive a car without adaptations

□ I drive an adapted car

□ Car driven by someone else

□ Taxi/Uber or equivalent

□ Ambulance

□ Other (please specify)

**53. How often do you have ALS follow-up appointments?**

□ Monthly

□ Every 3 months

□ Every 6 months

□ Yearly

□ Other (specify)

**54. How would you rate the ease of access to ALS follow-up appointments?**

□ Very easy

□ Easy

□ Neither easy nor difficult

□ Difficult

□ Very difficult

**55. How would you rate your sense of security (perceived support) regarding how you are supported in your disease (OUTSIDE the family context)?**

□ Completely secure

□ Secure

□ Neither secure nor insecure

□ Insecure

□ Very insecure (I do not feel supported)

**56. Please indicate which health professionals you have contact with in the context of ALS: (Select all that apply).**

□ Neurologist

□ Nurse

□ Physical therapist

□ Dietitian/Nutritionist

□ Occupational therapist

□ Social worker

□ Psychologist

□ Psychiatrist

□ Primary care/Family medicine physician

□ Speech-language therapist

□ Other (specify)

**57. Do you use any form of alternative medicine?**

□ Yes

□ No

**58. If “Yes,” which one(s)? (Select all that apply).**

□ Ayahuasca (ethnobotanical medicine)

□ Cannabis

□ Acupuncture

□ Reiki

□ Meditation

□ Osteopathy

□ Homeopathy

□ Other (specify)

**Nutritional Assessment Scores**

**MNA-SF—Mini Nutritional Assessment Short Form**

Score: ______/14

□ 12–14 points: Normal nutritional status

□ 8–11 points: At risk of malnutrition

□ 0–7 points: Malnourished

**MNA-FF—Mini Nutritional Assessment Full Form**

Score: ______/30

□ 24–30 points: Normal nutritional status

□ 17–23.5 points: At risk of malnutrition

□ <17 points: Malnourished

**GLIM—Global Leadership Initiative on Malnutrition**

GLIM score/stage: ______________________________

□ No malnutrition

□ Stage 1—Moderate malnutrition

□ Stage 2—Severe malnutrition
